# Recruitment of older adults with type 2 diabetes into a community-based exercise and nutrition randomised controlled trial

**DOI:** 10.1186/s13063-016-1589-5

**Published:** 2016-09-26

**Authors:** Eliza G. Miller, Caryl A. Nowson, David W. Dunstan, Deborah A. Kerr, Vicky Solah, David Menzies, Robin M. Daly

**Affiliations:** 1 Institute for Physical Activity and Nutrition, Deakin University, Geelong, VIC Australia; 2 Baker IDI Heart and Diabetes Institute, Melbourne, VIC Australia; 3 School of Public Health, Curtin University, Perth, WA Australia; 4 Fitness Australia, Melbourne, VIC Australia

**Keywords:** Recruitment strategies, Randomised controlled trial, Exercise, Nutritional supplementation, Type 2 diabetes, Older adults

## Abstract

**Background:**

Recruitment of participants into long-term community-based lifestyle intervention trials, particularly adults with a chronic disease, is often slow and challenging. Currently there is limited data on successful recruitment strategies suitable for older adults with type 2 diabetes into community-based exercise and nutrition programs, and no information on cost estimates associated with such recruitment. The aim of this report is to describe the recruitment strategies used and the success of each approach in recruiting older adults with type 2 diabetes into a 6-month community-based exercise and nutritional supplementation randomised controlled trial (RCT). A secondary aim is to assess the costs associated with the recruitment methods used.

**Methods:**

The Resistance Exercise, Vitamin D and Muscle Protein Intervention Trial (REVAMP-IT) for type 2 diabetes is a 24-week RCT targeting 202 adults with type 2 diabetes which is designed to evaluate whether post-exercise ingestion of a whey- protein and vitamin D-enriched drink can enhance the effects of progressive resistance training (PRT) on glycaemic control, body composition and cardiometabolic health. Participants in this trial were randomly allocated to either: (1) the *Lift for Life*® community-based PRT program combined with additional whey protein and vitamin D, or (2) the *Lift for Life*® PRT program alone. Recruitment strategies included state and local newspaper and radio advertisements, targeted mail-outs, doctor and allied health referrals, community presentations, web-based media and word of mouth. The number of expressions of interest, participants screened and included in the trial, and how they first heard about the study were recorded by research staff during the screening process. Reasons for ineligibility or non-participation in the trial were also recorded as was the cost of each recruitment method used.

**Results:**

A total of 1157 expressions of interest were received over a 21-month recruitment period. Overall 959 (83 %) individuals were screened and found to be ineligible for the trial or chose not to participate or could not be contacted further following their initial enquiry. As a result, 198 participants were randomised to the 24-week intervention. The most effective recruitment strategies were targeted mass mail-outs (39 % of the total participant sample), state (27 %) and local (14 %) print media. In total recruitment expenditure was AUD$40,421, which equated to AUD$35 per enquiry and AUD$204 per eligible participant. Targeted mail-outs and state print media were the most expensive strategies each accounting for 38 % of total expenditure.

**Conclusions:**

To recruit around 200 older adults with type 2 diabetes into a community-based lifestyle intervention trial in a timely manner, it is important to ensure that an adequate budget is allocated to recruitment as targeted mail-outs and state/local print media were the most costly but effective strategies.

**Trial registration:**

Australian New Zealand Clinical Trials Registry reference ACTRN12613000592741. Registered on 27 May 2013.

## Background

The success of randomised controlled trials (RCTs) is dependent on effective recruitment of the target population. One of the key challenges associated with conducting intervention trials, particularly those that target populations with specific medical conditions such as people with type 2 diabetes, is finding the appropriate balance between recruitment of an adequate number of participants within monetary budgets and established timelines. Failing to recruit the required number of participants can significantly impact upon the statistical power of the study and adversely affect the internal and external validity of the trial. Delays in recruitment can also extend the trial duration and result in an increase in trial and recruitment costs.

The Resistance Exercise, Vitamin D and Muscle Protein Intervention Trial (REVAMP-IT for type 2 diabetes) is a large-scale RCT involving 202 adults with type 2 diabetes designed to evaluate whether post-exercise ingestion of a whey-protein and vitamin D-enriched drink can enhance the effects of progressive resistance training (PRT) on glycaemic control, body composition and cardiometabolic health. Recruitment of older adults with type 2 diabetes into such a lifestyle modification trial can be particularly challenging because it often involves identifying those that must first meet certain inclusion criteria (e.g. sedentary lifestyle) and who are also motivated to change their behaviour (e.g. engage in regular exercise or change dietary habits). Despite compelling evidence that exercise and diet are key elements in the management (and prevention) of type 2 diabetes, individuals with this condition are typically inactive and have poor dietary habits [1, 2]. Therefore it is often difficult for these people to commit to a long-term behavioural change program that is central to these types of studies. Common barriers and reasons that have been identified for why many adults (including those with type 2 diabetes) do not participate in exercise trials include inconvenience, a lack of time to dedicate when managing work/family commitments and a lack of understanding of the potential health benefits of participating in such trials [3, 4].

A previous review of recruitment strategies for participants into RCTs revealed that methods which do not directly target the population of interest (e.g. type 2 diabetics), advertisements which are not effective in conveying the key objectives of the research or which fail to accurately inform of the potential personal health benefits to participation, can negatively impact on recruitment rates [5]. Currently there is limited data on successful recruitment strategies suitable for older adults with type 2 diabetes into community-based exercise and nutrition programs. One study has previously described the experiences and strategies used to recruit type 2 diabetic patients into an RCT to assess the efficacy of an automated, interactive voice-response telephone intervention to promote physical activity (PA) behaviour [4]. This study found that targeted mail-outs were the most effective method of recruitment compared to traditional recruitment strategies such as flyers and brochures which were posted and distributed in various healthcare settings and only provided a basic description of the study [4]. However, there still remains a large degree of paucity in data available describing successful recruitment strategies suitable for older adults with type 2 diabetes into community-based exercise and nutrition programs, and no information on cost estimates.

The aim of this report is to describe the recruitment strategies used and the success of each approach in recruiting older adults with type 2 diabetes into the 6-month community-based REVAMP-IT for type 2 diabetes exercise and nutrition research intervention in Melbourne, Australia. A secondary aim was to assess the costs associated with the recruitment methods used. Our goal was to recruit 202 adults aged 50 to 75 years into the study over a 12-month period. During the recruitment process, we tested a number of different recruitment strategies and documented the response data (and related costs) for each approach. By providing a detailed report of the recruitment experiences associated with this trial it is hoped this information will help to inform recruitment approaches for future intervention studies wishing to target older adults with type 2 diabetes into community-based lifestyle-related management trials.

## Methods/Design

### Study overview

A detailed description of the protocol for this RCT has previously been published [6]. Briefly, this study was a 24-week intervention in which participants were randomly allocated to either: (1) the *Lift for Life*® community-based PRT program combined with additional whey-protein and vitamin D supplements, or (2) the *Lift for Life*® PRT program alone. The primary outcomes were glycated haemoglobin (HbA_1c_) and homeostasis model assessment 2 (HOMA-2) of insulin resistance and beta-cell function. Secondary outcome measures included changes in: body composition, muscle strength, blood pressure, blood lipids, adipokines and inflammatory markers, growth factors, health-related quality of life and cognitive function.

All participants enrolled in the trial were asked to complete the *Lift for Life*® PRT program in local community health and fitness centres, which involved training twice a week for the first 8 weeks and three times a week thereafter. Training sessions were supervised by qualified trainers, and lasted approximately 45 to 60 minutes, and consisted of moderate to high intensity PRT (three sets of eight to ten repetitions) targeting all major muscle groups and with an emphasis on progressive overload (increments in weight of 2–10 % per week). All participants were charged a fee (AUD$3–$12) by their local health and fitness provider to undertake the program, but were eligible for reimbursement up to the value of AUD$240 at the completion of the study based on their level of compliance [6].

Those randomised to the *Lift for Life*® plus supplement group were also provided with a supply of a whey-protein-enriched powder and vitamin D supplements [6]. Participants were instructed to prepare and consume the whey-protein drink (20 grams of whey protein concentrate 80 % with 150 ml of cold water) every morning prior to breakfast and consume a subsequent whey-protein drink within 2 hours of each *Lift for Life*® training session. They were also required to consume two vitamin D capsules (1000 IU vitamin D_3_) each day. The PRT alone group received no additional powder or supplements.

Throughout the trial, participants were required to attend Deakin University for approximately 2 hours on two occasions (baseline and 24 weeks). Research staff conducted on-site testing at each health and fitness centre after 12 weeks. A rested morning (8–10 am) venous blood sample was additionally collected at an accredited pathology laboratory following an overnight fast on three occasions over the 24-week period (baseline, 12 and 24 weeks).

### Sample size calculations

As previously published [6], it was estimated that a sample size of 168 participants would provide the study with 90 % power (*P* < 0.05 two-tailed test) to detect a 0.5 % absolute difference for the change in HbA_1c_ levels between the groups, assuming a conservative standard deviation of 1.1 %. For insulin sensitivity, a sample size of 140 would be required to detect a 0.7 difference for the change in HOMA-2 insulin resistance between the groups at a power of 90 %, assuming a conservative standard deviation of 1.2. To compensate for an estimated 20 % drop-out rate, it was calculated that 202 participants would be recruited to the study and randomised 1:1 to either of the two groups (101 participants per group).

### Ethics

This trial was managed by staff within the Institute for Physical Activity and Nutrition (IPAN) at Deakin University Burwood, Melbourne, VIC, Australia and funded by a National Health and Medical Research Council Project Grant (ID1046269). The study was approved by the Deakin University Human Research Ethics Committee (HREC 2013-050), and is registered with the Australian and New Zealand Clinical Trials Registry (ACTRN12613000592741). All participants provided written informed consent prior to their commencement in the study.

### Screening and eligibility criteria

As reported previously [6], eligibility for this study was assessed using a two-step screening process. Briefly, participants were screened via a telephone questionnaire and ineligibility was based on the following criteria: (1) aged <50 or >75 years of age; (2) HbA_1c_ >10 %; (3) current or prior participation in a structured resistance training program >1 week or moderate-intensity physical activity ≥150 min/week in the previous 3 months; (4) vitamin D or calcium supplement use >500 IU/day and >600 mg/day, respectively, in the previous 3 months; (5) severe orthopaedic, cardiovascular or respiratory conditions that would preclude participation in an exercise program, or those with absolute contraindications to exercise, according to American College of Sports Medicine (ACSM) guidelines [7]; (6) renal impairment (estimated glomerular filtration rate (eGFR) <45 ml/(min 1.73 m^2^)) or disease; (7) regular use of protein supplements; (8) conditions that may affect vitamin D or calcium metabolism; (9) current smoker, or (10) body mass index (BMI) >40 kg/m^2^. Participants who passed the initial telephone screening were then required to obtain approval from their doctor to clear them of any medical conditions contraindicated to exercise, based on ACSM guidelines. Participants were also required to provide a fasted, morning blood sample to confirm that their HbA_1c_ level was <10 %.

### Recruitment strategies

During the trial establishment period, the research team met to develop a recruitment plan. The initial strategies adopted were similar to those which had been used previously by the trial staff in other lifestyle-related interventions. These included local newspaper advertisements, distribution of flyers/posters to relevant health professionals and community groups and GP presentations. Within the first 2–3 months, expressions of interest (EOIs) and recruitment of participants was modest, at which point the research team consulted with the Deakin University public relations and communications team to discuss the trial advertisements and communications approaches and how they could be improved. A number of new recruitment strategies, in addition to those already in use, were then implemented to recruit participants from metropolitan Melbourne and within a 50 km radius of Deakin University in Burwood, VIC, Australia as well as surrounding regional areas including Geelong, VIC, Australia. Such strategies included state and local newspaper and radio advertisements, targeted mail-outs through the National Diabetes Support Scheme (NDSS) member database, GP and allied health professional referrals, community presentations, web-based media and word of mouth. A brief overview of each of the approaches is presented below. For each participant, specific information relating to the method of recruitment was obtained during the initial screening (telephone) session.

#### State print media

A total of six paid advertisements were placed in leading state-wide newspapers, with the advertisements ranging in size, colour and placement within each paper. The content of these advertisements included an eye-catching headline, a brief and simple description of the trial, including the potential health benefits to participants associated with participation, and relevant contact details. The cost for these advertisements ranged from AUD$1700 to AUD$3000 each.

#### Local print media

Twelve paid advertisements, similar in format to the state-based advertisements, were also placed in a range of local municipality newspapers, which are delivered weekly to each household within a given area (municipality). In general, these advertisements were placed in the same paper fortnightly over a 6-week period, however, occasionally single advertisements were used to trial and target one locality to gauge the level of interest. The cost for the local paper advertisements ranged from AUD$400 to $500 each depending on the size and placement of the advertisement within the paper. A number of free advertisements were also placed in local newspapers in the community ‘What’s On’ section (limited to 40 words). These free advertisements were used as a buffer to continue the recruitment campaign between paid state and local media advertisements.

#### Radio advertising

The study was also advertised on a state-wide radio station. Forty-five, 15-second paid advertisements, which were drafted by study researchers and refined by the radio station, were played over a 5-day period (Monday to Friday). The radio station was selected based upon the demographics of the majority of their audience (e.g. middle-aged and older adults). The advertisements were initially played during the ‘Seniors Spotlight on Trials and Studies’ segment and thereafter were spread sporadically over the course of the day ranging from 6 am through to 10 pm. Advertising this trial on radio costs AUD$990 for the forty-five 15-second advertisements.

#### Targeted mail-outs

The research team gained approval from Diabetes Australia, a non-governmental organisation, to access the NDSS database, which is a federally funded register of Australians with diabetes that was initiated in 1987. Briefly, the aim of NDSS is to enhance the capacity of people with diabetes to understand and self-manage their condition, and to provide relevant information, support and access to essential products at subsidised prices. In Victoria, the NDSS register contains the contact detail of more than 85 % of all clinically confirmed cases of diabetes, which includes type 1, type 2 and gestational diabetes [8]. Since 2001, individuals joining the NDSS have been asked whether they would be willing to be informed of any opportunities to participate in research. Only those who consented to be contacted were eligible to be invited to participate in this study. Because of the extensive nature of the database, the research staff first used the online Australian Diabetes Map (http://www.diabetesmap.com.au/), which is an interactive national diabetes prevalence map established and regularly updated by NDSS and the Australian Bureau of Statistics (ABS), to identify local government areas and the postcodes within these areas with a reported high prevalence of individuals with type 2 diabetes. Thereafter, the research team contacted NDSS who were responsible for identifying the number of individuals within each of the identified postcodes that had type 2 diabetes, who were aged 50 to 75 years and reported that they were not currently taking insulin to manage their condition. A letter of invitation to participate in the study, that was prepared by the research staff but sent by NDSS, was then forwarded to all individuals that fulfilled the necessary inclusion criteria. Nine mail-outs were conducted and the number of letters sent per mail-out ranged from 80 to 5000 depending upon the number of registrants within a target area. The cost of a direct mail-out was approximately AUD$1.80 per letter. This cost increased or decreased marginally depending on the number of individuals contacted, e.g. the greater the number of letters sent the cheaper the cost. There were no additional costs associated with accessing the NDSS database.

#### Referrals from GPs and allied health professionals

General practitioners, endocrinologists and other relevant health professionals (e.g. diabetes educators, dietitians and pharmacists) identified from relevant directories (e.g. Yellow Pages), hospitals and profession-specific association websites who were based in a 50-km radius of Melbourne were sent information packs via the mail that provided details about the trial. Included in these packs was a one-page study summary, which outlined the aims, commitment required and the potential benefits to participants, and the key eligibility criteria. They also received trial flyers and posters and were asked to place these on notice boards and/or distribute them directly to relevant patients. Connections with several Medicare Local groups, which are regional organisations who co-ordinate improvements in healthcare for designated populations groups and oversee front-line primary healthcare services, were also made during the establishment of this trial. Specifically, the Diabetes Australia map was used to identify areas with a high prevalence of type 2 diabetes, and the respective Medicare Local groups were subsequently contacted to inform them of the trial and were provided with trial information and advertisements and requested to include these in any communications with GPs and allied health providers.

#### Flyers and presentations

Flyers and posters were placed in prominent public and relevant locations such as fitness clubs, libraries, senior citizen centres, pharmacies, community houses and retirement villages. In addition, three presentations were made to a range of audiences; one to a diabetes support group where the attendees were a mix of accredited Diabetes Educators, patients and carers, one to GPs at a Medicare Local meeting and another at a local Diabetes Educators annual general meeting.

#### Website advertisement

Information about the study was posted on the *Lift for Life*®, Diabetes Victoria, Diabetes Australia, Fitness Australia, Deakin University’s Institute for Physical Activity and Nutrition and the Australian Centre for Behavioural Research in Diabetes websites. These listings were placed on respective websites at the beginning of the recruitment campaign and requested to be removed from the website at the completion of recruitment. There was no cost associated with posting information on these websites.

#### Social media

A pay-per-click advertisement (AUD$0.03 per click at a capped limit of AUD$500) was placed on the social media domain Facebook. This advertisement was 20 words long and used key words to capture potential participant’s attention. The advertisements appeared on an individual’s Facebook page whose age fell into the eligible age range for the trial and a postcode pre-identified to be within a 15-km radius of Deakin University. An individual who clicked on an advertisement was directed to the *Lift for Life*® website for further information about the trial. The cap of AUD$500 was not reached during the recruitment period but did allow for approximately 16,600 clicks to the advertisement.

#### Word of mouth

Participants enrolled in the trial were actively encouraged to refer family, friends or colleagues with type 2 diabetes into the study. In addition, participants with type 2 diabetes who enquired but were ineligible for other research studies being conducted within IPAN and who had consented to be contacted for any other research studies, were contacted by research staff via telephone to determine their interest and eligibility in regards to participating in this trial.

### Data collection and analysis

The number of expressions of interest (EOIs), participants screened and included in the trial, and how they first heard about the study was recorded by the research staff throughout the screening and recruitment process. Reasons for ineligibility or non-participation in the trial were also recorded. These were categorised into one of five categories: health-related, physical activity status, medication/supplement use, travel or age. Response rates were reported as the number and percentage of participants both interested in and recruited into the trial. Recruitment yield was calculated as the total number of participants recruited divided by the number of EOIs. The success of each recruitment strategy was determined from the number of participants recruited divided by the number of EOIs from each strategy expressed as a percentage. Recruitment rates were also calculated as the average number of participants recruited and randomised per month throughout the recruitment phase. Results were also split by gender. The cost of each recruitment method used was calculated by dividing the total amount spent on a given strategy by: (1) the number of EOIs generated for each strategy, and (2) the total number of eligible participants the strategy was responsible for recruiting (e.g. cost per participant recruited).

## Results

### Recruitment

Recruitment for this trial was intended to be completed within 12 months. This was extended to a period of 21 months (February 2014 to October 2015) due to a lower than expected recruitment rate, particularly during the initial stages. In total, 1157 EOIs were received from which 198 older adults with type 2 diabetes were deemed eligible for the trial and subsequently randomised. This represents 98 % of the original specified target sample size of 202 participants.

### Response rates, eligibility and reasons for ineligibility or non-participation

Of the total 1157 EOIs received for this trial, 198 (17 %) older adults with type 2 diabetes were deemed eligible for the trial and subsequently randomised. Overall 516 (45 %) individuals screened were found to be ineligible for the trial and a further 443 (38 %) chose not to participate or did not respond following their initial enquiry. As shown in Table 1, the most common reasons for ineligibility included being too physically active (11 %), currently taking vitamin D supplements at a dose of >500 IU/day (11 %) and management of type 2 diabetes by way of insulin therapy (5 %). A further 189 (16 %) participants indicated that they were no longer interested in participating in the trial upon discussing the study requirements with the research staff, 62 (5 %) reported that they could not meet the financial obligations of committing to the PRT program, 61 (5 %) did not live within a practical travel distance to Deakin University or have a training facility within their immediate area, and 4 (1 %) chose not to participate as they did not wish to take supplements of any form. A further 127 individuals (11 %) could not be contacted following their initial enquiry.

**Table 1:** Number and proportion of participants deemed eligible and ineligible for the study and the reasons for ineligibility or non-participation

		Total, n (%)
Eligible		198 (17 %)
Ineligible		516 (45 %)
Other reasons for non-participation		443 (38 %)
Total screened		1157 (100 %)
Reasons for ineligibility or non-participation	*Health-related*	115 (10 %)
	▪ Not diagnosed with type 2 diabetes	*28 (2 %)*
	▪ Any medical condition contraindicated to resistance exercise	*18 (2 %)*
	▪ BMI >40 kg/m^2^	*30 (3 %)*
	▪ HbA_1c_ >10 %	*14 (1 %)*
	▪ Presence or indication of kidney disease	*8 (1 %)*
	▪ Current smoker	*17 (1 %)*
	*Physical activity status*	135 (11 %)
	▪ Meeting/exceeding current physical activity guidelines	*135 (11 %)*
	*Medication or supplement use*	192 (17 %)
	▪ Prescribed insulin for management of type 2 diabetes	*59 (5 %)*
	▪ Taking vitamin D supplements >500 IU/day	*128 (11 %)*
	▪ Taking dietary protein supplements	*5 (1 %)*
	*Travel*	53 (5 %)
	▪ Travel plans	*53 (5 %)*
	*Age*	21 (2 %)
	▪ Aged <50 or >75 years of age	*21 (2 %)*
	*Other*	443 (38 %)
	▪ Unable to afford the *Lift for Life®* program	*62 (5 %)*
	▪ Remoteness to Deakin University or PRT training facility	*61 (5 %)*
	▪ Did not wish to take supplements of any form	*4 (1 %)*
	▪ Did not wish to participate	*189 (16 %)*
	▪ Could not be contacted for screening following first enquiry	*127 (11 %)*

Presence or indication of kidney disease was considered if estimated glomerular filtration rate (eGFR) <45 mL/min. Potential participants were excluded if meeting or exceeding National Physical Activity Guidelines of 150 minutes of moderate to vigorous physical activity or participating in greater than one strength-training session per week. Participants were excluded if they responded yes to taking dietary protein supplements at a protein content of >10 g per serve as specified on the products nutrition information panel

*BMI* body mass index, *HbA1c* glycated haemoglobin *PRT* progressive resistance training

### Recruitment return by recruitment strategies

As shown in Table 2, targeted mail-outs to NDSS members were the most successful approach for recruitment, generating 479 (41 %) of the 1157 EOIs, from which 78 participants were eligible for the trial (39 % of those randomised). State-wide free and paid advertisements generated the second greatest number of enquiries (n = 326, 28 % of total), from which 54 participants were eligible and subsequently randomised. Advertising in local newspapers (both paid and unpaid) generated 173 enquires (15 %), with 27 of these enquiries deemed eligible. Website advertisements produced 96 enquires (8 %) that led to 19 participants randomised. All other forms of recruitment generated a low number of enquires and resulted in only 20 eligible participants in total: word of mouth (n = 6, 3 %); referral from health professionals (n = 10, 5 %); flyers/presentations (n = 4, 2 %), with no eligible participants from radio advertising and social media.

**Table 2:** Number and proportion (percentage) of expressions of interest and participants deemed eligible for the trial based on the various recruitment strategies

	Eligibility status			
	Eligible		Expressions of interest	
Recruitment strategy	n	%	n	%
Targeted mail-outs	78	39	479	41
State print media	54	27	326	28
Local print media	27	14	173	15
Website advertising	19	10	96	8
Allied health referrals	10	5	19	2
Word of mouth	6	3	37	3
Flyers/community presentations	4	2	11	1
Radio advertising	0	-	6	0.5
Social media	0	-	5	0.4
No record	0	-	5	0.4
Total	198	100	1157	100

### Recruitment return by gender and age

**Fig. 1:**
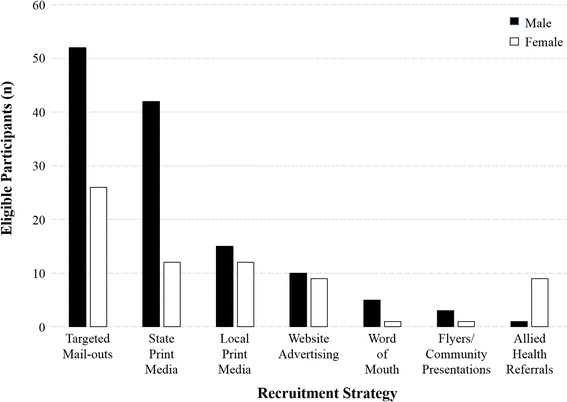
Number of male and female participants eligible for this trial according to the recruitment strategies utilised

The total number of males screened and recruited into the trial exceeded females [males: screened n = 669 (58 %) and eligible n = 128 (65 %); females: screened n = 488 (42 %) and eligible n = 70 (35 %)]. Figure 1 shows the number of male and female participants eligible for this trial according to the different recruitment strategies utilised. The only recruitment strategy that resulted in a higher number of females (n = 9) than males (n = 1) was allied health referrals, but the total number of referral from this method was very low. In terms of the age distribution, 39 % of all eligible participants were aged 50–59 years, 53 % were aged 60–69 years with the remaining 11 % aged over 70 years.

### Recruitment timeline

**Fig. 2:**
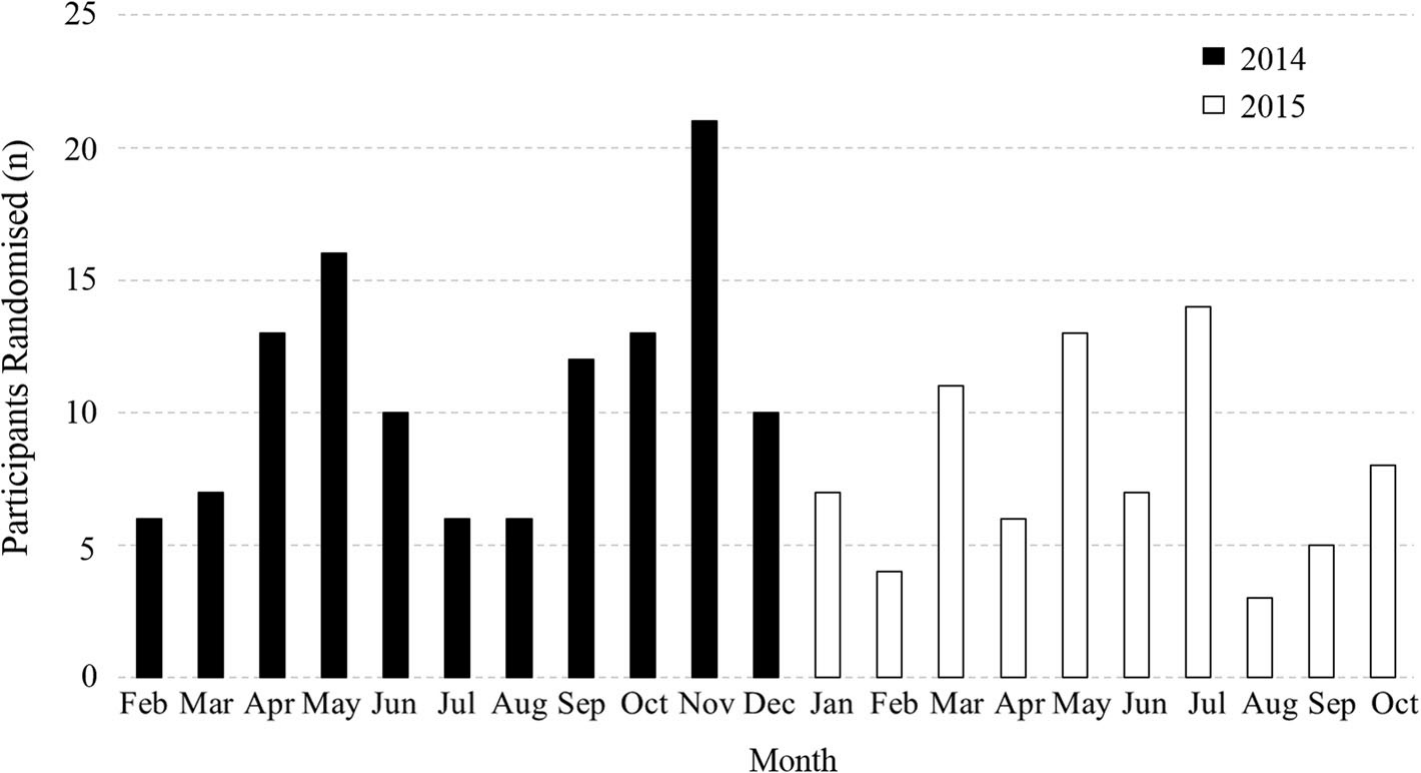
Number of participants randomised to the trial each month for the duration of the recruitment period

Figure 2 illustrates the number of participants randomised per month over the 21-month recruitment period. On average, nine participants were randomised per month. The peaks on the graph correspond with the implementation of state-based newspaper advertisements (April, May, August, June, November 2014 and February 2015) and targeted mail-outs through the NDSS (September, October and December 2014 and May, July, September and October 2015). Not all strategies were implemented continuously throughout the campaign, however, several methods of recruitment were often in place at the same time.

### Recruitment cost

Table 3 provides recruitment yield by cost for each recruitment method. Expenses included in the cost analysis do not include personnel costs (two full-time staff were employed to work on the project) as they were not tracked by each method and were not able to be differentiable from other tasks performed in the administration of the trial. The most costly recruitment methods were both state print media and NDSS mass mailing, which both accounted for 38 % of the total recruitment budget. Overall the total costs attached to these two recruitment strategies were AUD$15,360 and AUD$15,327 respectively. Cost per eligible participant was greatest for state print media at AUD$284. By comparison the higher yield of eligible participants from the NDSS mass mailing meant the cost per eligible participant was lower at AUD$196. Costs per eligible participant for local print media were similarly to that for state print media ($AUD239). Printed (paid) trial flyers and posters, which were used at community presentations and provided to allied health professionals, was the cheapest form of paid recruitment, but also generated the least number of eligible participants. Radio advertising and social media were not found to be effective methods of recruitment for this trial as no eligible participants were recruited from these approaches. Free methods of recruitment including focused website advertising and word of mouth were responsible for generating 25 eligible participants for the trial. The total recruitment costs for this trial were AUD$40,421, which equated to AUD$35 per enquiry and AUD$204 per eligible participant.

**Table 3:** The total costs, proportion of total recruitment costs, the number of eligible participants plus cost per participant for each recruitment strategy used in this trial

	Eligible participants (n)	Cost per eligible participant^a^	Total cost^a^	% of total recruitment costs
Targeted mail-outs	78	$196	$15,327	38
State print media	54	$284	$15,360	38
Local print media	27	$239	$6,445	16
Allied health and flyers/presentations	14	$128	$1,799	4.5
Radio advertising	0	-	$990	2.5
Social media	0	-	$500	1
Website advertisement	19	$0	$0	0
Word of mouth	6	$0	$0	0
Total	198		$40,421	100

^a^Personnel costs are not included in this analysis. All costs of recruitment are in Australian Dollars

## Discussion

In this 6-month community-based exercise and dietary supplementation trial in older adults with type 2 diabetes, a total of 1157 EOI were received over a 21-month recruitment period from which 198 participants (17 %) were deemed eligible and subsequently randomised. These figures reflect an average randomisation rate of nine participants per month. The most effective recruitment strategies for this trial were targeted mail-outs to registered members of the NDSS database (39 % of the total sample) and state print media (27 %). Overall, recruitment expenditure totalled AUD$40,421, with the NDSS and state print media being the two most expensive (and effective) recruitment strategies. In total, recruitment costs equated to AUD$35 per enquiry and AUD$204 per eligible participant. Collectively, these findings highlight the significant challenges accompanying recruitment of older people with type 2 diabetes into a community-based lifestyle modification trial.

### Eligibility rate

Of the 1157 EOIs, only 198 participants were found to be eligible, equating to a 17 % success rate. This rate of recruitment was similar to the 19 % recruitment rate reported in the Look AHEAD (Action for Health in Diabetes) trial [9]. The aim of this large multi-centre RCT was to investigate the effects of a lifestyle intervention aimed at reducing body weight by 7 % through increased physical activity (PA) and a reduction in calorie intake on a range of health outcomes, including incidence of heart attacks, stroke and cardiovascular-related death in overweight or obese adults with type 2 diabetes [10]. Comparing the recruitment success rates of different trials can be problematic as factors such as differences in the inclusion/exclusion criteria will influence recruitment rates. Nevertheless, other exercise intervention trials recruiting sedentary overweight and obese post-menopausal females have reported recruitment rates ranging from 2 to 20 % [11–14]. Thus the recruitment rate in our trial appears to be consistent with previous intervention trials in older adults and provides a realistic target for future exercise and/or nutrition-based RCTs designed to target older adults with type 2 diabetes, particularly where the design of the trial has similar eligibility criteria and involves a comparable level of commitment.

### Success of recruitment strategies

The recruitment strategies used in this study were diverse and varied depending on their success as the recruitment phase evolved. Mass mail-outs through the NDSS was the most successful strategy, being responsible for recruiting 39 % of all eligible participants. While it is difficult to compare the success rate of our mass mail-out campaign to other trials given the varying eligibility criteria, our rate is slightly above that reported in the Diabetes Prevention Program (DPP) conducted in the United States, where 29 % of its randomised participants were recruited via direct mass mail-outs into their multi-centre RCT aimed at discovering whether modest weight loss through dietary changes and increased physical activity or treatment with oral diabetes medication could prevent or delay the onset of type 2 diabetes in those at high risk for the condition [15]. The ability of mass mail-outs to target a large number of participants at a relatively low cost, combined with the reported success of this strategy to yield a high number of eligible participants in both healthy and clinical populations, has in recent years led to this approach becoming an integral part of many recruitment plans [4, 16–18]. From our experience, when implementing mass mail-outs as a recruitment strategy it is the choice of the mail-out lists which are likely to contribute to its effectiveness. For instance, in our trial we were able to selectively request the NDSS target members of the registry who met three of our inclusion criteria [type 2 diabetes diagnosis, current management of condition (prescribed only oral hypoglycaemic medication if any) and age]. The NDSS database also allowed letters to be sent only to local government areas and postcodes which trial staff had identified as areas with a high prevalence of type 2 diabetes. We believe that this selective communication to a tailored list of potential participants in specific areas greatly assisted in the efficient identification of eligible participants.

State and local print media were the second and third most prolific methods of recruitment, yielding 27 % and 14 % of participants, respectively. Other RCTs using state and local print media have reported similar recruitment rates from such advertisements [15, 19, 20] and previously it had been reported that print media, including direct mail-outs plus newspaper advertisements, are two strategies which are more effective at capturing the attention of an older adult demographic [15]. It has also been reported that the most efficient and comprehensive recruitment campaigns employ several overlapping strategies simultaneously, with constant assessment of the strategies employed to ensure appropriate allocation of resources to the most successful approaches [21]. In our study, the research staff remained diligent tracking and assessing the effectiveness of each recruitment strategy with only those with the greatest yield of eligible participants persisted with. This may have resulted in the success rate of the mass mail-outs and state media being over-reported as these strategies were employed repeatedly compared to those that resulted in a smaller and slower yield of eligible participants (e.g. web advertising, allied health referrals, word of mouth, flyers and community presentations).

Interestingly, medical and allied health referrals were not successful in recruiting older adults with type 2 diabetes into the trial. Similar findings were reported in a large community-based Australian vitamin D supplementation trial in older adults and the elderly [16]. In our trial, it cannot be determined whether this was due to medical or allied health professional’s lack of communication about the trial, poor distribution of trial advertising material and/or if there was a general lack of interest in participating in an exercise program from individuals attending these appointments. Based on our experiences it is a recommendation for future trials to consider practice-wide presentation sessions to patients, nurses and interested clinicians which eliminates the burden on health professionals to convey trial information and may result in a greater percentage of trial participants recruited from this approach.

### Cost

In total, AUD$40,421 was spent on all recruitment strategies combined, which equates to an average of AUD$204 per eligible participant. Very few detailed cost analyses of other large-scale RCT recruitment campaigns exist making it difficult to comparatively assess the methods and associated costs of recruiting eligible participants. However, a trial investigating weight gain prevention in young adults reported the average cost of recruiting one participant was US$233 in 2014 [17]. Similarly, a study investigating the recruitment of minority women into the Women’s Health Trial, which also utilised targeted mass mail-outs, reported per participant recruitment costs ranging from US$100 to $144 in 1998 [22]. In contrast, the DPP trial which randomised 3819 participants spent on average US$1075 per randomised participant [23]. The recruitment strategies employed by the DPP trial make comparing costs to our trial difficult as the DPP employed a public relations agency to help direct recruitment who worked alongside a recruitment committee with their recruitment phase spanning a total of 3 years and costing $US4,105,000 [24].

Despite state print media acquiring the second highest number of eligible participants for our trial, it was the most expensive method, costing AUD$15,360 or AUD$284 per eligible participant. Local print media advertising costs were considerably lower than state media however, the small yield in eligible participants (14 %) resulted in this method being the second most expensive recruitment strategy. Mass mail-outs through the NDSS produced a high eligibility to response ratio resulting in this approach being implemented multiple times over the recruitment period. Whilst this approach was the second most expensive strategy used (AUD$15,327) and accounted for 38 % of the total dollars spent on recruitment, the large yield of eligible participants resulted in this approach being one of the most cost effective at AUD$196 per participant.

### Reasons for ineligibility and choosing not to participate

Of all the EOIs received for our trial, 45 % (516 participants) were deemed ineligible according to the inclusion/exclusion criteria. Of these, 11 % was due to the participants being classified as too physically active (e.g. meeting or exceeding the Australian National Physical Activity Guidelines). This result is not unexpected as there is recent evidence that approximately 30 % of adults with type 2 diabetes were found to meet physical activity guidelines [1]. A finding which is much smaller compared to another recent RCT recruiting type 2 diabetes patients to an automated, interactive voice-response telephone intervention to promote physical activity behaviours in adults with type 2 diabetes, which excluded 26 % of potential participants due to physical activity levels [4].

Reasons for non-participation in RCTs for people that initially enquire are frequently missing from recruitment publications, but is important to consider as these factors may offer valuable insight into areas of trial design that may be improved to enhance recruitment to future trials. In our trial, 189 (16 %) potential participants felt the requirements of the trial (training) was too much of a commitment and did not wish to participate. A further 62 individuals or 5 % of the EOIs received cited not being able to bear the financial costs (approximately AUD$300–700 dependent upon on training site) associated with joining the *Lift for Life*® program, despite the incentive of a monetary reimbursement at the completion of the trial based upon each participant’s level of compliance to the exercise program. Unfortunately, no information was collected on the potential effects of the monetary reimbursement acting as an incentive to participate in the trial. Similarly, the effect that having to pay for the *Lift for Life*® training program had on people’s reluctance to participate in the trial was also not assessed.

### Limitations

There are a number of limitations that should be considered when interpreting the findings from this report. Our study specifically targeted older adults with type 2 diabetes for an exercise and dietary supplementation trial and so the results and experiences reported should be tempered when applying to other trials recruiting alternate clinical populations. The effects of the monetary reimbursement on incentive to participate in the trial were not assessed and future studies need to ask these types of questions to assess the effects of such incentives on willingness to participate in research. The recruitment strategies found to be most successful in sourcing eligible participants for this trial were also the ones which were repeated multiple times potentially biasing the findings. A more reliable assessment of success or failure of recruitment strategies would be one where the number of eligible participants recruited is expressed proportional to the individuals exposed to a strategy. Unfortunately, we have no way of quantifying exactly how many individuals viewed our state and/or local newspaper advertisements, listened to our radio advertisements or viewed our trial listing on a website. Therefore we have had to depict the success rate based upon those eligible for the study from the total who enquired from a given strategy. Furthermore, similar messages were used for all of the advertisements and as such no data is available for what type of message may be most salient to older adults with type 2 diabetes. Additionally, only one recruitment strategy was recorded per expression of interest leaving open the potential for cross-contamination from different recruitment methods. For example, some participants who received a mail-out may also have been exposed to a trial advertisement in web or print media format, however, with only one form of recruitment noted for each expression of interest the effects of exposure to two recruitment strategies remains unknown.

## Conclusions

This report aimed to address the practical considerations surrounding recruitment of older adults with type 2 diabetes into a 24-week lifestyle modification intervention. Recruitment required continual sustained efforts and necessitated flexibility within the recruitment strategies used to ensure the recruitment targets were being achieved. Of the vast array of recruitment approaches employed, strategies which were found to be most effective were targeted mail-outs to members of the NDSS database and state print media. The least effective strategies were radio advertising and social media. Overall, AUD$40,421 was spent on recruiting participants to this trial, at a cost of AUD$204 per randomised participant. This does not include personnel costs. Overall, these findings provide a unique insight and offer several lessons and future directions related to recruiting type 2 diabetes patients into trials involving lifestyle modification.

### Trial status

Recruitment and data collection have been completed and data analysis has commenced.

## Abbreviations

DPP: Diabetes Prevention Program
EOI: expression of interest
HbA_1c_: glycated haemoglobin
HOMA-2: homeostasis model assessment 2
Look AHEAD: Action for Health in Diabetes
NDSS: National Diabetes Support Service
PA: physical activity
PRT: progressive resistance training
RCT: randomised controlled trial
